# Control of nutrient metal availability during host-microbe interactions: beyond nutritional immunity

**DOI:** 10.1007/s00775-023-02007-z

**Published:** 2023-07-18

**Authors:** Karrera Y. Djoko

**Affiliations:** Department of Biosciences, Durham University, Durham, DH1 3LE UK

**Keywords:** Nutritional immunity, Host-microbe interactions, Microbes, Metal buffering, Metal availability

## Abstract

The control of nutrient availability is an essential ecological function of the host organism in host-microbe systems. Although often overshadowed by macronutrients such as carbohydrates, micronutrient metals are known as key drivers of host-microbe interactions. The ways in which host organisms control nutrient metal availability are dictated by principles in bioinorganic chemistry. Here I ponder about the actions of metal-binding molecules from the host organism in controlling nutrient metal availability to the host microbiota. I hope that these musings will encourage new explorations into the fundamental roles of metals in the ecology of diverse host-microbe systems.

Earth is home to a trillion of species of microbes [1]. Some of these microbes associate with larger, more complex living organisms (hosts). These host-associated microbes (termed “symbionts”) include mutualists that benefit the health and development of their hosts, commensals that neither benefit nor harm the host, and parasites (or pathogens) that harm the host. These symbionts often establish complex communities on host epithelial tissues and are collectively known as the “microbiota”.

What is the function of the host in host-microbiota relationships? Ecological models posit that hosts construct a nutritional landscape that differentially affects different symbionts [2, 3]. This host function is relatively well described for carbohydrates. For example, mammalian gut tissues produce fucosylated glycans (fucans), which are consumed by *Bacteroides* [4] (a gut mutualist) but not *Salmonella* [5] (a gut pathogen). Although *Roseburia* (another gut mutualist) cannot use fucans, it can use dietary mannans [6]. Perhaps it is no surprise that carbohydrates are often highlighted as major drivers of microbiota assembly, dynamics, and stability [7–12].

What if different microbial symbionts use the same nutrient? For example, metals are used universally by all symbionts, since these micronutrients are needed by almost half of all enzymes [13], including in microbes. Mutualists, commensals, and pathogens all likely need Zn to carry out transcription [14] and translation [15]. Or, perhaps they all require Fe and/or Cu to respire and produce energy [16, 17]. Metals can and do become the limiting nutrient and thus drive inter-microbial competition in the host [18, 19]. However, all metals are also microbial poisons when in excess. Thus, shifts in the total levels of a *single* metal inside a host can differentially starve, feed, or poison different microbial symbionts, depending on each symbiont’s individual metal needs and tolerances.

A quick PubMed search reveals numerous studies involving dietary metal restriction or supplementation in human [20–23], animal [24–30], and even insect [31] hosts. These studies almost invariably conclude that changes in the host’s metal nutrition influence the microbiota [23, 32]. But do the host organisms act as mere vessels for metals and microbes? Or do host organisms play a role in mediating the effects of metals to their microbiota?

A wealth of evidence from the last half century, particularly from studies with mammalian hosts, indicates that hosts *do* mediate shifts in metal levels, specifically to *suppress pathogens*. [33] In response to infection or inflammation, *diseased* hosts produce metal-binding effectors that change metal speciation and, thus, *availability* (Fig. 1A). This host action is, of course, known as “nutritional immunity” (a term first coined in 1973) [34], and it is conserved in diverse hosts, including vertebrates [35], invertebrates [36], and plants [37].

**Fig. 1:**
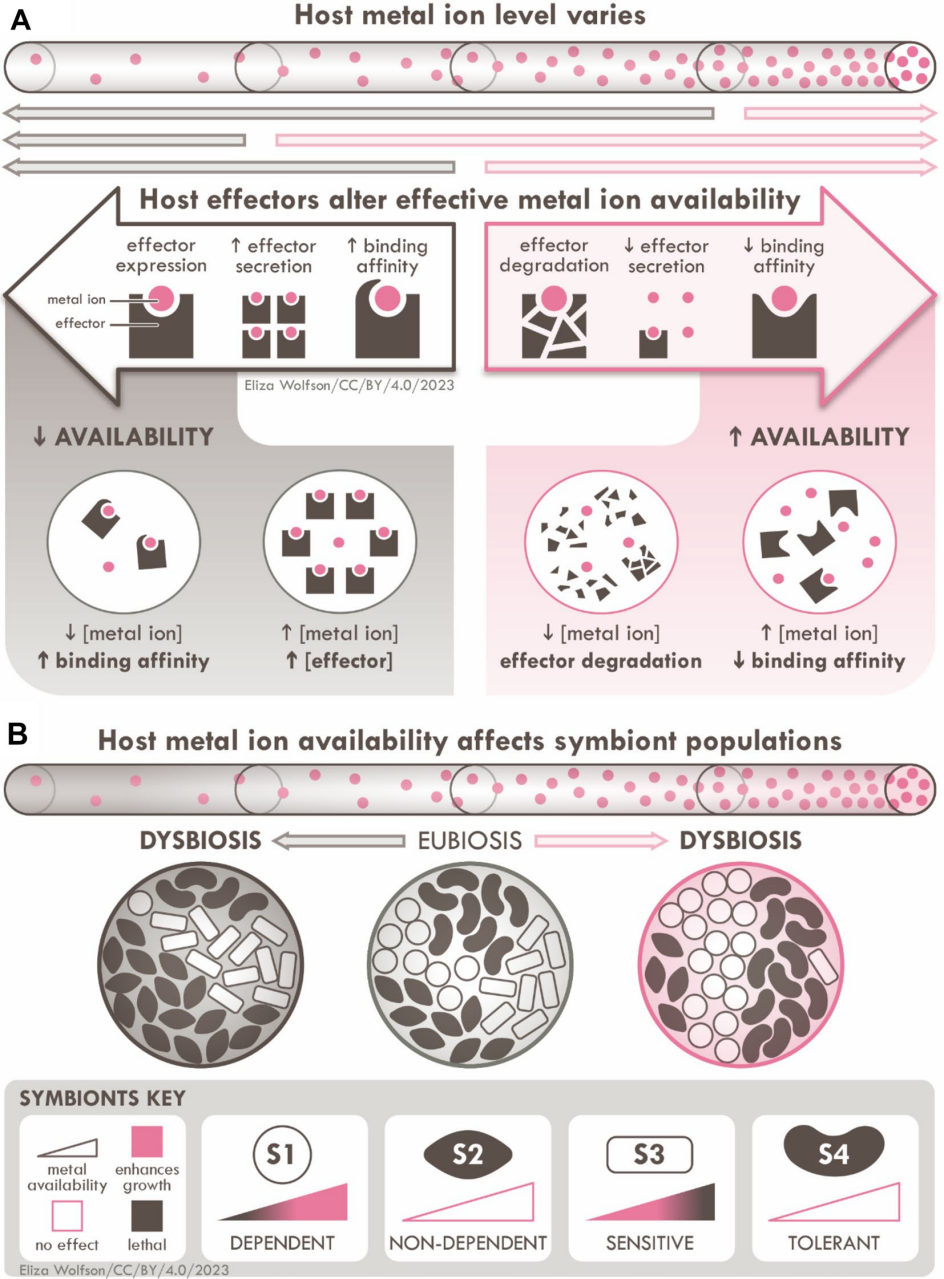
Metal availability in the host influences the health of host-associated symbionts. **A** Host metal-binding effectors mediate metal availability in host niches. As explained in the main text, metal availability can change depending on total metal levels, total effector levels, and the binding affinity of the effector to the metal. **B** Host metal availability differentially affects the health of different host-associated symbionts, depending on each symbiont’s nutritional needs and tolerances. The specific scenarios involving symbionts S1–S4 are described in the main text

The overall picture that has emerged over these last 50 years is that nutritional immunity promotes microbial *clearance* from the host. Most known host effectors sequester metals, reduce metal availability, and starve the pathogen (e.g. calprotectin and Zn or Mn [35], transferrin and Fe). Other effectors appear to bring more metal ions to the site of inflammation, raise metal availability, and poison the pathogen (e.g. ceruloplasmin and Cu [38]).

Do hosts also mediate shifts in metal levels, not to suppress pathogens, but to *support mutualists (and commensals)*? Despite the pathogen-centric view of host-microbe interactions in the metals in biology literature, the overwhelming majority of host-microbe interactions is, in fact, *not* pathogenic. Do *healthy* hosts also produce metal-binding effectors to promote microbial *colonisation* (instead of clearance)? Instead of nutritional immunity, can host effectors maintain supply of metal nutrients and simultaneously limit metal toxicity to microbes?

It may be useful to first hypothesise what happens in the absence of such an effector. Consider two symbionts, S1 and S2. Although the general concept likely applies to other host niches, for simplicity, let us imagine that both symbionts colonise the host gut. Let us also assume that the basal level of a particular metal in that host gut is such that S1 and S2 can usually cohabit. However, S1 needs this metal as a nutrient to support metabolism, while S2 does not. Without a host effector, increases in total metal levels and, thus, availability, which can occur during host feeding, can preferentially promote growth of S1, potentially allowing S1 to outcompete S2 (Fig. 1B). Conversely, decreases in total metal levels and availability, which can occur during host fasting, can preferentially restrict growth of S1, potentially allowing S2 to grow and fill the niche (Fig. 1B).

For completion, let us consider two other gut symbionts, S3 and S4. S3 is sensitive to metal toxicity while S4 is tolerant. Without a host effector, increases in metal levels and availability can preferentially suppress growth of S3, potentially allowing S4 to grow and fill the niche. Conversely, decreases in metal levels and availability can preferentially permit growth of S3, potentially allowing S3 to outcompete S4 (Fig. 1B).

Regardless of the identity of each symbiont (whether mutualist, commensal, or pathogen), all scenarios above depict microbiota that are susceptible to *disturbance*. Such disturbances, termed “dysbiosis”, *i.e.* the overgrowth or loss of one or more symbionts (Fig. 1B), can have adverse consequences to host health and development.

However, if there exists a metal-binding host effector that binds and *buffers* the metal sufficiently strongly, then such disturbances can be avoided. By buffering the metal, this effector would prevent large swings in metal availability upon increases or decreases in metal levels (Fig. 2A). This host effector would allow the microbiota (and, in turn, the host) to maintain homeostasis over a much wider range of total metal levels. In our hypothetical scenarios earlier, the host effector suppresses metal supply to S1 and S3 during host feeding, but maintains this supply during host fasting. This scenario now depicts a stable and healthy host microbiota, which is termed “eubiosis” (Fig. 1A).

**Fig. 2:**
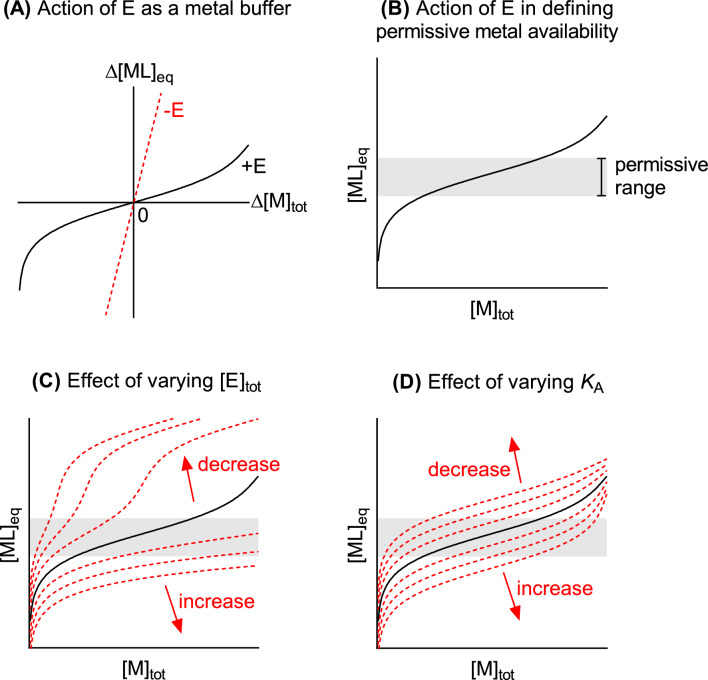
Model for how a host metal-binding effector E buffers a nutrient metal M to symbionts. The relevant reaction equilibria are shown for: (1) Weak and non-specific binding of metal M by ligand L. In this model, the metal–ligand ML complex represents the available (kinetically and/or thermodynamically exchangeable) pool of metal in the host. The identity of ligand L is unknown but is likely to comprise combinations of metal-binding molecules that originate from either the host, the symbionts, or their external environment. Levels of ligand L are likely undefined and subject to uncontrolled fluctuations. (2) Strong and specific binding of metal M by host effector E. Thus, metal M that is bound in the metal-effector ME complex is considered less available (or less readily exchangeable). The identity of effector E is presently unknown. Unlike Ligand L, effector E originates from the host and thus its levels are likely better defined or controlled. (3) Competition between ligand L and host effector E for binding metal M. Only 1:1 ML and ME complexes are shown for simplicity, although higher order complexes (ML_2_, ME_2_, etc.) are possible. **A** Action of the host effector E as a strong metal buffer, suppressing large swings in metal availability (Δ[ML]_eq_) in response to changes in total metal levels in the host (Δ[M]_tot_). **B** A host effector E, present at a particular total concentration and binds metal M at a particular binding affinity, defines the metal buffering capacity of the host and thus the permissive range of metal availability ([ML]_eq_) for symbionts. **C** Effects of increasing or decreasing the total concentrations of host effector E ([E]_tot_) on host metal availability ([ML]_eq_). **D** Effects of increasing or decreasing the binding affinity of the host effector to metal [*K*_ME_; see Eq. (2)] on host metal availability ([ML]_eq_)

The laws of thermodynamics and inorganic chemistry predict that metal availability varies with effector concentration (Fig. 2C) and metal-binding affinity (Fig. 2D). Thus, a host effector that binds a metal ion with a particular affinity and is present at a particular concentration will, in essence, define the upper and lower boundaries of metal availability in the host (Fig. 2B), within which different symbionts can assemble and stabilise. Any symbiont can, in principle, colonise the host if it possesses metal needs and tolerances that are compatible with metal availability in the host. If host metal availability is higher than what is tolerated or lower than what is needed by a symbiont, then that symbiont will fail to colonise the host.

Now consider a pathogen, whose needs and tolerances are, incidentally, compatible with metal availability in the host. In response to inflammation by this pathogen, metal availability can, presumably, be altered by the host (Fig. 1A). For example, the local effector concentration can be altered by varying effector expression, secretion, or degradation. Or, metal binding to the effector can be tightened or weakened by post-translational modification of the effector at or near the metal-binding site. These actions can either lower or raise metal availability beyond the normal range (Fig. 2C and D) and thus suppress growth of the pathogen. If this seems familiar, it should, because this is what happens as a result of nutritional immunity.

But won’t nutritional immunity also adversely affect growth of mutualists (and commensals)? Yes, it likely will. Prolonged inflammation, at least in humans, is indeed associated with dysbiosis of the microbiota [39]. It is not unlikely that nutritional immunity contributes to this process, by promoting microbial metal starvation and/or microbial metal poisoning in a sustained manner.

What host molecules can act as the hypothesised effectors during healthy host-microbe interactions? *Any* extracellular or secreted metal-binding molecule from the host can presumably achieve this function, as long as its metal affinity is relatively high and its background concentrations in the relevant host environment are relatively stable (*cf.* Figure 2C). Obvious candidates include components of the polydisperse layer of mucus that coats host epithelial tissues, on which symbionts typically reside. For example, mammalian gut mucins [40] and human salivary histatins bind Cu(II) [41]. Do they buffer Cu availability to the mammalian gut and human oral microbiota? Alternatively, the host effectors may be the *same molecules* that are already known to participate in nutritional immunity in *diseased* hosts. Perhaps *healthy* hosts secrete these molecules at much lower concentrations than those that promote microbial clearance. For example, calprotectin is usually detected in healthy stool samples, indicating basal secretion of this host effector into the gut. In addition, there is evidence that calprotectin helps regulate the assembly of the gut microbiota in infants [42]—does this involve differential supply of Zn or Mn to different gut microbes? Or, given the sheer diversity of host-microbe systems, perhaps many of these host effectors are entirely novel and yet to be discovered.

To test the ideas outlined here, at least initially, my research group is in the process of establishing an experimentally tractable microbial community, composed of microbes whose metal needs and tolerances differ, so that growth of each microbe is differently influenced by varying total metal levels. In the absence of the naturally relevant host effector, increasing or decreasing total metal levels should encourage some microbes to become dominant in the community, while others become diminished. However, addition of the host effector should allow this community to maintain its composition more successfully. These steps certainly describe an investigation in microbial ecology, but one that is rooted undeniably and firmly in the principles of bioinorganic chemistry.
